# The reproductive strategy of a typical distylous *Ophiorrhiza alatiflora* (Rubiaceae), in fragmented habitat

**DOI:** 10.3389/fpls.2024.1492402

**Published:** 2024-11-04

**Authors:** Yu Li, Ren-Xiu Yao, Bo Xu, Yun-Jing Liu, Bai-Zhu Li, Ming Tang, Yin Yi, Zhi-Rui Wen, Xiao-Yue Wang, Xiao-Xin Tang

**Affiliations:** ^1^ Key Laboratory of National Forestry and Grassland Administration on Biodiversity Conservation in Karst Mountainous Areas of Southwestern China, College of Life Science, Guizhou Normal University, Guiyang, China; ^2^ College of Resources and Environmental Sciences, China Agricultural University, Beijing, China; ^3^ Engineering Research Center of Carbon Neutrality in Karst Areas, Guizhou Normal University, Guiyang, China; ^4^ School of Life Sciences, Southwest University, Chongqing, China; ^5^ School of Life Sciences, Central China Normal University, Wuhan, China; ^6^ Guizhou Collaborative Innovation Center of Green Finance and Ecological Environment Protection, Guizhou University of Finance and Economics, Guiyang, China; ^7^ School of Chemistry, Chemical Engineering and Biotechnology, Nanyang Technological University, Singapore

**Keywords:** distyly, pollination, *Ophiorrhiza alatiflora*, incomplete self-incompatibility, ancillary polymorphic, habitat fragmentation

## Abstract

**Introduction:**

Heterostyly is a genetically controlled style polymorphism, that plays an important role in promoting outcrossing and improving reproductive fitness. Although distyly is often studied in plants of the Rubiaceae family, little attention has been paid to the reproductive strategies of distylous species in fragmented habitats. Here, We report for the first time the growth of *Ophiorrhiza alatiflora*, a type distylous species, in karst areas and evaluate its reciprocity between long styled morph and short one. We analyze the two distyly morph differences in the ancillary polymorphic of flowers and explore their reproductive strategy in fragmented habitats.

**Methods:**

In this study, we measured the floral characteristics of different morphs and performed differential secondary metabolite analysis on different morphs and tissue organs; Different pollination treatments were carried out to observe the fruit set, pollen germination, and pollen tube elongation of *O. alatiflora*.

**Results and discussion:**

Our research indicates that *O. alatiflora* is a typical distylous plant for the distyly has high reciprocity. Both morphs exhibit the highest fruit set of intermorph outcrossing; The pollen germination and pollen tube elongation experiments have also demonstrated that the affinity of pollen from intermorph outcrossing is highest, regardless of whether it is the long or short morph as the maternal parent; Meanwhile, *O. alatiflora* is an incompletely self-incompatible plant that exhibits a certain degree of self-pollination and intramorph outcrossing, which may be one of the important means to ensure sustainable reproduction in severely disturbed habitats. In the ancillary polymorphic of flowers, L-morphs flowers produce more pollen, and S-morph flowers produce more ovules to improve their male-female fitness and compensate for the asymmetry of pollen flow; Compared with S-morphs, L-morphs contain significantly higher levels of several kinds of terpenoids. S-morphs produce more flavonoids than L-morphs. The differences in secondary metabolites between L-morphs and S-morphs are mainly reflected in the different nutritional organs (including stems and leaves). Overall, our work has revealed the unique reproductive strategy of *O. alatiflora* in fragmented habitats based on the characteristics of distyly, verifying the hypothesis that the distyly of *O. alatiflora* promotes outcrossing and avoids male-female interference, improving male-female fitness and this is the first time in the *Ophiorrhiza* genus.

## Introduction

Angiosperms cover the entire spectrum from the hermaphrodite condition (female and male sexual organs within the same flower) to dioecy (female and male sexual organs in flowers of different individuals) (Barrett, 2002). In some monoecious plants, heterostyly as a unique flower structure that involves two or three morphs, the spatial separation of anthers and stigma allows pollen to have a greater chance of being released from one flower to another as a new flower than within the same flower, which is called outcrossing, and can provide plants with opportunities for rich genetic diversity and reduces the impact of inbreeding decline, promoting plant reproductive diversity and being an important sexual reproductive strategy developed by flowering plants during their evolution. But at the same time, if plants face adversity, they sometimes also choose to increase their chances of self-pollination to prioritize their survival needs.

For a long time, heterostyly has been considered one of the most effective mechanisms to promote outcrossing (Hernandez-Marquez et al., 2022). Darwin (1877) held that the reciprocal position of sexual organs between morphs could promote disassortative pollen transfer because morph-specific pollen segregation into distinct parts of the pollinator’s body should favor legitimate pollen deposition on L- and S-morph stigmas, this intermorph pollination is called legitimate and the intramorph as illegitimate. The breeding system of heterostyly, which is highly compatible with outcross pollination (legal pollination), and self-incompatible (illegal pollination), is unique and is called the heterotypic self-incompatibility system (Darwin, 1877; Bremer and Manen, 2000).

However, the efficiency of heterostyly in promoting disassortative pollen transfer has been questioned due to the presence of a significantly high proportion of illegitimate pollen on stigmas of several species (Nishihiro et al., 2000; Barrett and Shore, 2008; Brys and Jacquemyn, 2010; Liu et al., 2015). In fact, according to previous reports, heterosexual plants rarely exhibit perfect reciprocity (Faivre and McDade, 2001; Keller et al., 2012; Brys and Jacquemyn, 2015). During the interaction between pollinators and plants, complex factors such as changes in pollinator types and behaviors, as well as variations in plant floral organs, often result in asymmetric transfer of legal and non-legal pollen (Wolfe and Barrett, 1989; Brys et al., 2008). In addition, many studies have shown that plant self-incompatibility is mainly controlled by an allele located at the S locus, rather than solely caused by heterosexuality (McCubbin and Kao, 2000). Some researchers have proposed heterostyly, possibly to avoid interference between males and females. The precise heterosexuality of the pistils and stamens in most distylous species is conducive to the differentiation of male and female functions of sexual organs, allowing pollinators to deliver inter-pollen more accurately, reducing intra-pollen deposition, improving inter-pollination efficiency, and promoting male and female fitness (Lloyd and Webb, 1986; Castro et al., 2004).

With the continuous expansion of research directions, it has been found that distylous species not only differ in typical characteristics such as reciprocal herkogamy of male and female organs, but also have some trend in other aspects such as corolla size, pollen grain morphology, quantity, stigma morphology, and stored substances in pollen among different morphs. These characteristics are collectively referred to as ancillary polymorphic. The differences in ancillary polymorphic between different morphs may be the result of the interaction between plants and their environment, for example, some S-morph corollas are slightly larger than L-morph corollas; L-morph have a higher pollen content but slightly smaller pollen grains, while S-morph single flower has a lower pollen content but larger pollen grains; The S-morph stigma has more and smaller papillae, etc. To explain the reproductive characteristics of *O. alatiflora* from multiple perspectives, in addition to conventional pollination experiments, we also focused on the differences in ancillary polymorphics between two morphs, including differences in floral traits, and differences in secondary metabolites of nutritional and reproductive organs (Dulberger, 1992; Barrett and Shore, 2008; Ganguly and Barua, 2020).

The chemical composition in plants is a metabolic product produced by plant growth, development, and adaptation to the environment. Studying style heterostyly plants from the perspective of secondary metabolites is beneficial for elucidating the metabolic differences among different germplasm, as well as the effects of germplasm and environment on secondary metabolism. Besides, the abundant secondary metabolites of plants play an important role in influencing the behavior of pollinators. Complex mixtures of secondary metabolites can exhibit synergistic positive or negative effects. Flavonoids, anthocyanins, and carotenoids affect plant color, while terpenoids, amines, and phenylpropanoids contribute to unique odors. Clear color and odor markers are more conducive to pollinator discovery (Wink, 2008, 2018). In addition to attracting effective pollinators, plants may deter negative pollinators or herbivores with secondary metabolites. Dipsacus saponin, a defense compound, is present in the pollen of two *Dipsacus* species. The bitter taste of this substance makes bumblebees not comb the pollen, which is more conducive to the flow of pollen as male gametes, instead of being wasted as food (Wang et al., 2019b). Glucosinolates in vulgaris have the function of defending against herbivorous insects, while other plants in the same genus also contain toxic terpenoids to resist being eaten (Hussain et al., 2019). It is currently unclear whether secondary metabolites have a distinct way of balancing biased pollination in heterostylous plants. Further research is needed to fully understand the role of secondary metabolites in the relationship between plants and pollinators. We analyzed the differences in secondary metabolites of nutritional and reproductive organs between different morphs, hoping to identify typical differential metabolites that play an important role in pollinator attraction or defense processes. This is a novel and beneficial attempt in the study of heterostylous plants.

Currently, heterotypic have been reported in 193 genera and 30 families among them, the distribution of distylous species is pervasive, with at least 26 families of plants exhibiting distyly, especially in flowers with tubular and radiative symmetry. Among them, the Rubiaceae family has the most distribution, and more than half of the genera in this family have distyly (Lloyd, 1992; Chen et al., 2010). We observed in the field of Yunnan Province that several populations of *Ophiorrhiza alatiflora* H.S.Lo grow naturally on both sides of the road. To our knowledge, there are few reports on dimorphism within the genus *Ophiorrhiza*, and even few studies have explored the reproductive strategies of distylous plants in fragmented habitats, particularly in karst areas. Research in this area is crucial for understanding the adaptive evolution of plants. Karst regions are characterized by rugged terrain, severe erosion caused by flowing water, diverse and complex landforms, fragile ecological environments, and widespread habitat fragmentation (Wang et al., 2019a). In specific research areas, changes in ecological factors may be minimal. Most studies on the adaptability of karst plants to complex ecological factors—such as drought, high temperatures, and elevated calcium levels primarily focus on physiological regulation. For instance, *Lonicera confusa* adapts to drought by altering the distribution of stomata on the leaf epidermis. Additionally, the upper parts of *P. tatarinowii* can mitigate damage from high temperatures and intense light in karst environments by increasing photosynthetic yields while reducing respiration (Liu, 2021). However, there has been limited discussion on the evolutionary reasons for the survive of these plants in such unique environments, and the influence of habitat fragmentation on plant adaptation and survival has often been overlooked. In addition to changes in ecological factors across different regions, habitat fragmentation resulting from human activities or complex geological processes may significantly influence the selection of reproductive strategies during plant evolution, particularly for distylous plants in karst areas experiencing considerable habitat fragmentation. Numerous studies have shown that habitat fragmentation is a primary factor leading to the loss of biodiversity and species extinction, and it is also a significant threat to the survival of natural organisms (Berger, 1990; Rodríguez and Delibes, 2003; Gentili et al., 2018) Habitat fragmentation not only affects species richness but may also disrupt gene flow between populations, leading to inbreeding, decreased or lost genetic diversity, and greater challenges to individual survival. Consequently, habitat fragmentation has become a focal point of international biodiversity conservation research (Didham et al., 1996; Heinken and Weber, 2013). It remains uncertain whether *O. alatiflora*, the subject of this study, is also experiencing the negative effects of inbreeding decline due to habitat fragmentation. We aim to explore the adaptation strategies of distylous plants to habitat fragmentation in karst areas from an evolutionary perspective.

In addition, the extended *O. alatiflora* exhibit typical heterostyly with two morph symbiosis, mainly pollinated by *Lasioglossum* sp. and *Bombus* sp. In theory, plants in fragmented habitats may increase self-pollination to cope with unfavorable living environments, but on the other hand, the abundance of pollinators in this habitat provides a favorable prerequisite for plant outcrossing. Our question regarding this is:(1) What is the reproductive strategy of *O. alatiflora* and how does it strike a balance between preserving offspring resources and avoiding inbreeding decline? (2) As a typical distylous plant, what are the differences in ancillary polymorphic between different morphs of *O. alatiflora*?

We hope to evaluate the degree of mutual deviation between different morphs and attempt to explain the reproductive strategy of *O. alatiflora* in fragmented habitats from the perspectives of the breeding system and ancillary polymorphic, providing new clues for the origin and evolution of heterostylous plants. Specifically, this study mainly carried out the following work: (1) evaluated the degree of reciprocal hermaphroditism between different morphs; (2) By conducting artificial pollination experiments and pollen germination experiments, evaluate the potential for pollen germination and fertilization under different pollination treatments, and determine the main reproductive strategies of *O. alatiflora*. (3) By analyzing the ancillary polymorphics of different morphs, we aim to elucidate the contribution of differential ancillary polymorphic characteristics in the adaptive evolution process of *O. alatiflora*.

## Materials and methods

### Study species and site

*O. alatiflora* is an herb belonging to the Rubiaceae, native to China, and discovered in calcareous soil in Yunnan provinces. Its corolla is white with purple stripes and has a downy tube, five anthers enclosing a bifid stigma. *O. alatiflora* was a distylous plant with L-morph (with anthers low in the corolla and high stigmas) and S-morph (with anthers high and stigmas low).

**Figure 1:**
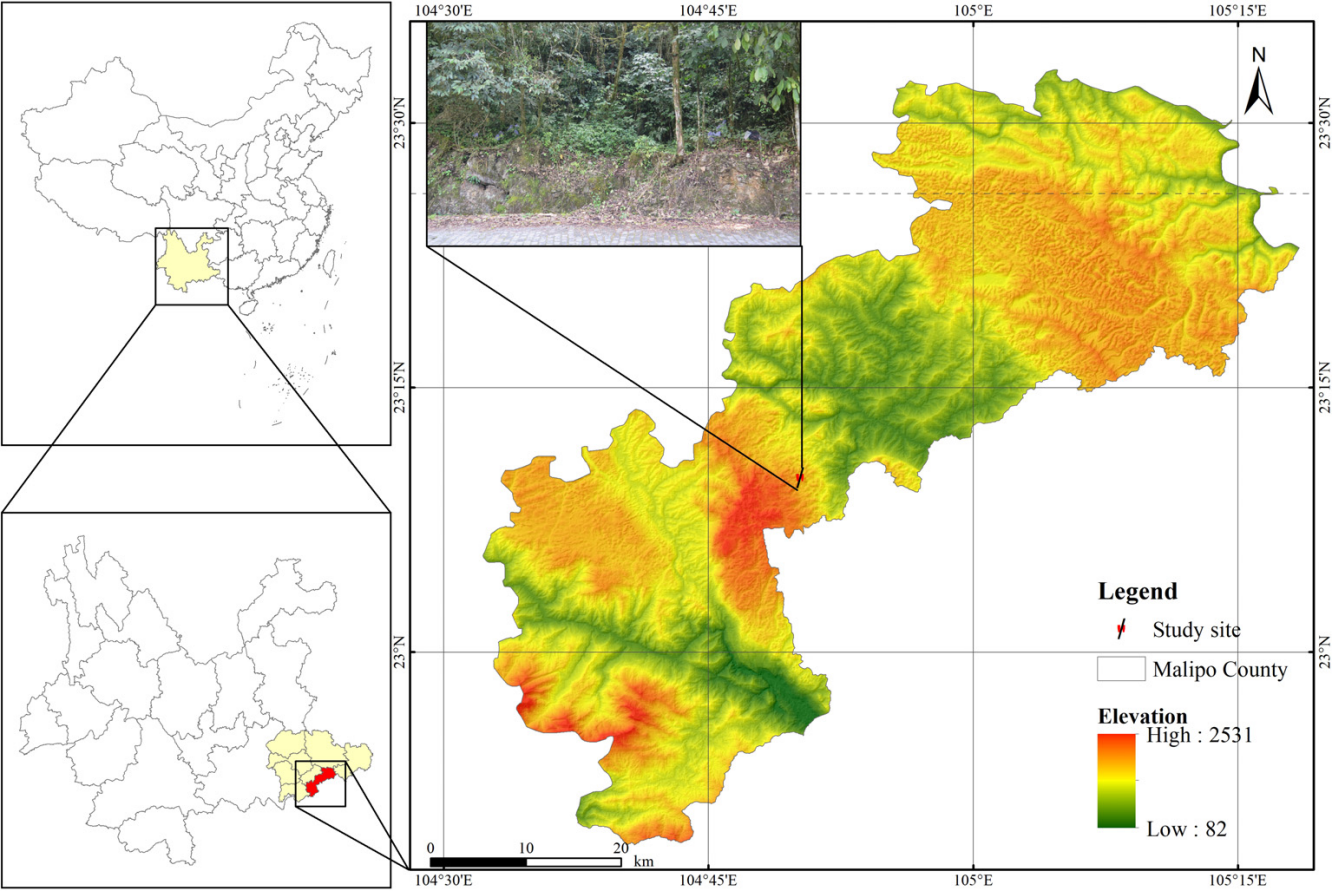
Schematic diagram of the study site.

We conducted the field experiments at Laoshan Provincial Nature Reserve (23°9’52.97” N,104°50’12.04” E; approximately 1551 m) in Malipo county, Yunnan province, southwest China during April and June 2021 (**Figure 1**). We obtained the *O. alatiflora* plants with the permission of Laoshan Provincial Nature Reserve.

### Pollinator species and abundance

To determine the effective pollinator of *O. alatiflora*, we observed all visits of different species for seven sunny days (April 21-30) in 2022. Each session lasted for 30 min. we randomly chose about 50 blooming flowers per morph. When one visitor came, the visit number per foraging bout, visitor species, and foraging behavior (for the nectar or pollen) were recorded. The visit frequency of one visitor was equal to the mean number of visits per flower per hour. If the visiting insect extended its long tongue into the corolla tube and touched the anthers (removing pollen grains) and stigmas (depositing pollen grains), it was considered an effective pollinator. The species and behavior of visitors were recorded using cameras (Nikon D7500). After that, we captured the visitors made them into specimens, and brought them back to the laboratory for identification.

### Pollen germination and breeding system

To determine whether *O. alatiflora* is self- and intramorph incompatible, we randomly selected at least 30 healthy flowers for bagging in each treatment and conducted artificial pollination treatment when these flowers were fertile: (1) open pollination as control; (2) intramorph pollination: L-morph as pollen receptor receiving L-morph pollen from other individuals and S-morph as pollen receptor receiving S-morph pollen from other individuals; (3) intermorph pollination: L-morph as pollen receptor receiving S-morph pollen from other individuals and S-morph as pollen receptor receiving L-morph pollen from other individuals; (4) self-pollination: pollen from the flowers of the same individuals; (5) autogamy treatments: the flowers were bagged all the time without any treatments; and (6) emasculated treatments. Except for the control, all treatments were bagged to discourage insect access and prevent visitors. Two months after pollination, fruits of the six pollination treatments were collected and counted. Due to multiple uncertainties in the field, not all the treated fruits could be collected. However, the sample size for all the pollination treatments was greater than 30.

To test the difference in pollen germination and pollen tube growth between the four treatments (above treatment 1 - 4) of the two morphs. Pollination experiments have shown that autogamy and emasculated treatments of *O. alatiflora* result in a very low fruit set, so these two treatments are not considered in the following experiments. Pollination treatments (1 - 4) were conducted between 09:00 and 10:00 h. Pistils of these flowers were collected 6 hours later. Each was preserved in FAA solution (for malin – acetic acid - 75% alcohol, 5: 5: 90 by volume) in a centrifuge tube. The pistil underwent three washes with distilled water and was then softened in 8 mol/L NaOH solution for 4 hours. After three more rinses with distilled water, the pistil was stained for 4 hours with 0.1% aniline blue, as described by Wang et al. (2018). Under a fluorescence microscope, we observed germinated pollen grains on the stigmas and pollen tubes in pistils and counted the total number of pollen grains on the stigma and the number of germinated pollen grains. Pollen tubes were first photographed and then the length of the pollen tubes was measured with the Image analyzer software (Digimizer Version 4.6.0) (Wang et al., 2018).

### Floral traits

To compare floral traits between the L-and S-morphs, we randomly chose 15 plants per morph, selected two flowers from each plant, and measured two vegetative traits, including leaf length and width, and fifteen reproductive traits, comprising flower length and width, petal length and width, tube depth and diameter, opening diameter, stamen length, pistil length, and anther and stigma length to 0.01 mm using a caliper micrometer. We labeled one floral bud on each of 20 different plants in 2022. When they bloom 24 hours later, record the life span of a single flower at 5 p.m. every day.

Calculate the interaction index of high trait height (L-morph pistils and S-morph stamens) and low trait height (L-morph stamens and S-morph pistils), RH and RL are denoted respectively. Among them, the high-level sex organ interaction index R_H_=(AP_H_-SP_H_)/(AP_H_+SP_H_), and the low-level sex organ interaction index R_L_=(AP_L_-SP_L_)/(AP_L_+SP_L_). The subscripts H and L represent high and low sex organs, respectively; AP_H_ and SP_H_ are the average stamen length of S-morph flowers and the average pistil length of L-morph flowers, respectively; AP_L_ and SP_L_ are the average stamen length of L-morph flowers and the average pistil length of S-morph flowers, respectively. R_H_ and R_L_ values vary from -1~1, with 0 representing better interactivity. The heterostyly is |R| (|R_H_| or |R_L_|) < 0.05 (Richards and Koptur, 1993; Ayiguli et al., 2021).

To compare pollen and ovule production, we selected 30 L-morph flower buds and 30 S-morph flower buds from different individuals and stored them in 1.5 mL centrifuge tubes filled with 75% alcohol. In the laboratory, the anther and ovary from one flower bud per plant were separated using forceps, and the anthers were completely crushed with tweezers to obtain 1.5 mL of pollen suspension. Three 15 μL drops of each pollen sample were counted, and the mean was multiplied by 100 to estimate pollen production. Pollen grains from these anthers were counted under a light microscope (Nikon E100). The ovules were counted under a stereomicroscope and P/O based on pollen count and ovule count (Cruden and William, 1977).

### Nectar traits

To compare the nectar volume and concentration between the two morphs, we labeled and bagged 60 flowers before anthesis (one from each of the 30 plants of each morph). The nectar of the marked flower was removed using glass microcapillary tubes (0.3 mm in diameter) on the day before the measurement. After 24 hours, we measured the length (L) of the microcapillary tube containing nectar using a caliper micrometer and calculated the volume (V_total_) and length (L_total_) of one standard microcapillary were calculated, and the volume of nectar (V) is equal to L/L_total_*V_total_. The concentration of nectar was measured using a handheld refractometer (Eclipse 0 ~ 50%; Bellingham and Stanley Ltd., Basingstoke, United Kingdom) and expressed as g of sugar per 100 g of solution (Corbet, 2003).

### Secondary metabolites

To investigate the difference in secondary metabolites between L-morph and S-morphs, five plants were randomly selected for sampling, and 3-5 buds per plant were bagged respectively. Until the anthers were mature and secreted nectar, three pollen samples were respectively collected with tweezers and three nectar samples with microcapillary tubes and placed in filter paper strips in 1.5 mL centrifuge tubes and stored in -20°C. The stem, leaf, and flower tissues were preserved at room temperature in a sealed plastic Ziplock bag with silica gel desiccant. They were then dried in an electric blast drying oven (Model 101-2AB, Tianjin Tester Instrument Co. Ltd.) for drying (70°C, 24 h) before further use.

Three samples of stem, leaf, and flower each were well ground using a mill (Nail, AQ-180E-X) and sieve repeatedly until there were no large solids, then the 9 samples were weighed 0.01 g using a balance (Sartorius BAS124S) and 1 mL methanol was added. Three pollen samples, each weighing 0.01 g, were treated with 1 ml of methanol using an ultrasonic homogenizer. The lyophilized nectar was dissolved at room temperature and weighed at 0.01 g. Then 1 mL methanol was added to the nectar sample and it was extracted at room temperature for 48 hours. After centrifugation at 10,000 rpm for 5 minutes, the supernatant (750 μL) was retained (Wang et al., 2019b).

The issue extracts were analyzed by Ultra Performance Liquid Chromatography (ACQUITY UPLC H-class, Waters) with Flow Through Needle (FTN) sample manager - Quadrupole/time-of-flight mass spectrometers (Xevo G2-XS QTof, Waters). Acquisition mode: ESI+, ESI-, MS. Acquisition range: 50-1500 Da (Scanning time 0.1s). 20μL extracts liquids were injected onto C18 column (ACQUITY UPLC BEH, 1.7μm, 2.1*100 mm, 40°C). Samples were eluted with solvents A, H2O (with 0.01% formic acid); B, Acetonitrile (with 0.01% formic acid) with the following program: A = 95%, B = 5% at 0 min; A = 95%, B = 5% at 2 min; A = 2%, B = 98% at 17 min; A = 2%, B = 98% at 20 min. The flow rate was 0.4 mL/min. The data acquisition was obtained by the UNIFI scientific information system (Waters). The components were reviewed by comparison with the Chinese traditional medicine database (Waters) with good-match analyses. The chemical formula and response value of each chemical compound were tentatively identified. The higher the chemical substance content in the examples, the larger the response value. The response value represents the relative content of each chemical substance in each gram of sample.

### Data analysis

To assess the differences in plant performance between L- and S-morphs, we compared plant vegetative and reproductive traits, flower lifetimes and pollen/ovule ratio, nectar volume, and sugar concentration using a generalized linear model (GLM) with normal distribution and identity-link function. The pollen grain number and ovule number were compared between L- and S-morphs using Poisson distribution with log-linear-link function in GLM (all plant characters as dependent variable, and L- and S-morphs as factors). Data of visits were analyzed using GLM with normal distribution and identity-link function (visitation rates as dependent variables, and flower morphs and visitor types as factors) to compare the visiting rates (visits/flower/hour) of all visitors between the two morphs.

PCA and Volcano plot was performed on the relative content of secondary metabolites (data of the detected secondary metabolites and their relative contents) using the MetaboAnalyst 5.0. Based on the volcano plot results, the obtained multivariate analysis of P value and fold change (FC) can initially screen out the different varieties of secondary metabolites. *P* < 0.05, fold change (FC) ≥ 2 and fold change ≤ 0.5 were used to select differently expressed metabolomics (DEMs).

To assess the differences in reproductive success between L- and S-morphs, we compare the pollen tube length of different treatments (pollen tube length as dependent variable, morphs, pollination treatments, and flower morph as factors). Pollen germination rates and fruit set of different treatments were examined with binary logistic analysis in GLM (full fruit number as event variable, total fruit number as trait variable, and pollination treatments and flower morph as factors). All data were analyzed in SPSS 26.0 (IBM Inc., New York, NY) software, we used Origin (2021) to generate the graphs.

## Results

### Species and frequency of flower visitors

**Figure 2:**
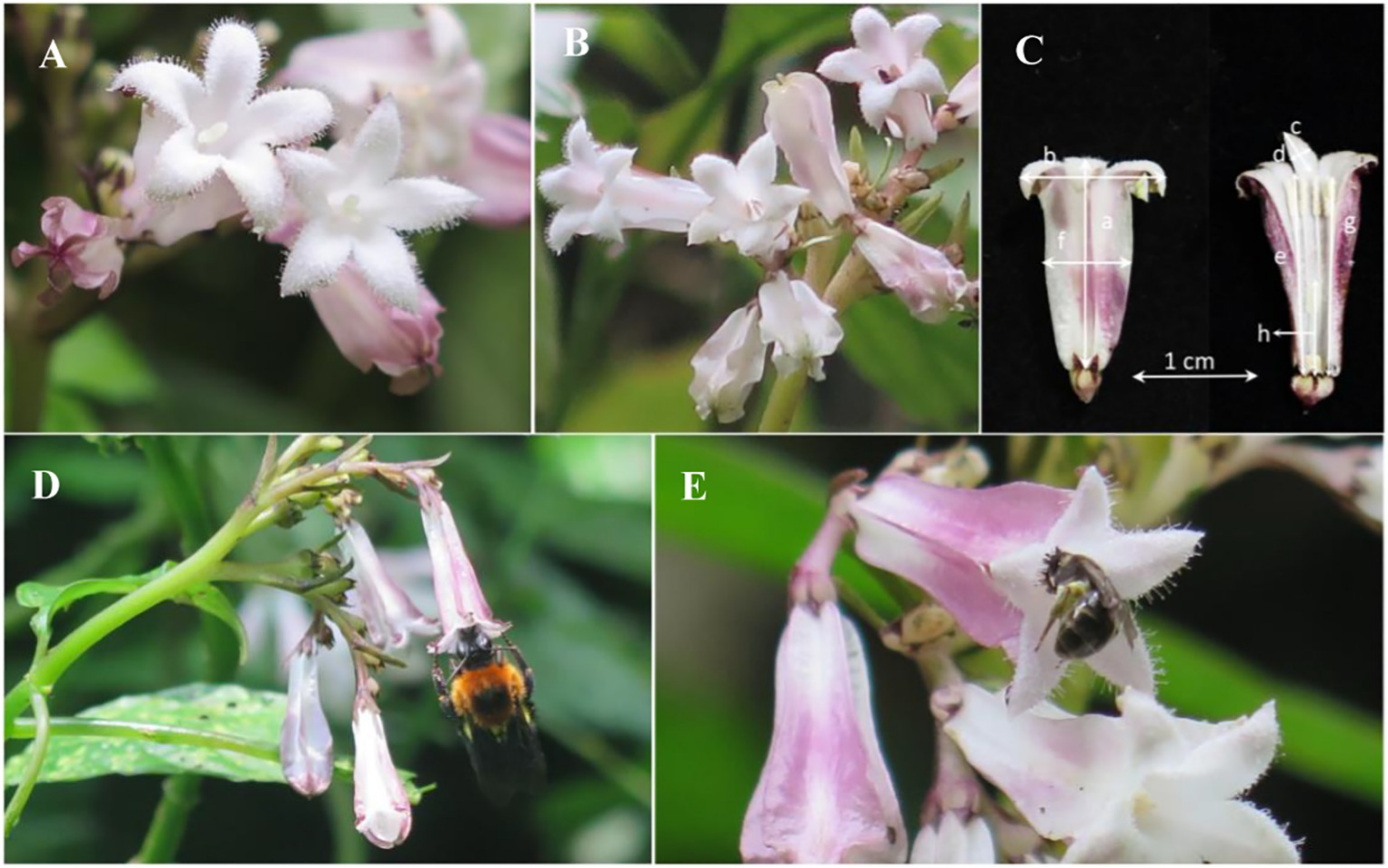
Floral traits and main visitors of *O. alatiflora*. **(A)** Inflorescence of L-morph flowers. **(B)** Inflorescence of S-morph flowers. **(C)** Measurement of floral traits of distylous *O. alatiflora* (S-morph as an example), a, Flower length; b, Flower width; c, Petal length; d, Petal width; e, Tube depth; f, Tube diameter; g, Stamen length; h, Pistil length. **(D)** *Bombus* sp. are visiting the flowers of *O. alatiflora*. **(E)** *Lasioglossum* sp. are visiting the flowers of *O. alatiflora*.

**Figure 3:**
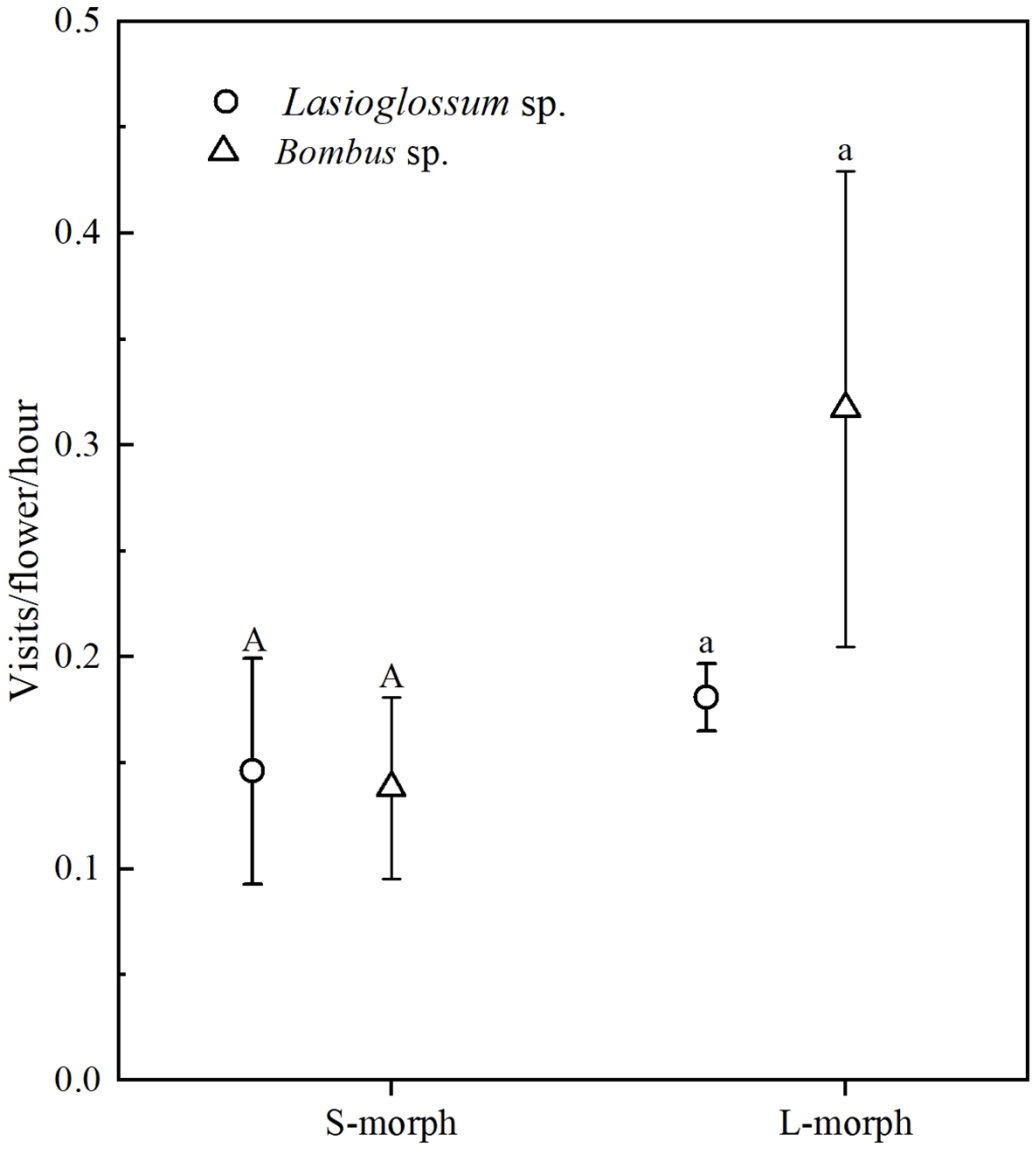
The comparison of visits/flower/hour between *Lasioglossum* sp. and *Bombus* sp. to L- and S-morphs of *O. alatiflora*. Bars sharing the same letters are not significantly different in visit rates between the two visitors.

We observed the flower visitors of *O. alatiflora* from April 21 to April 30, 2022, and found that the main visitors were *Bombus* sp. and *Lasioglossum* sp. (**Figure 2**). According to statistics, among the S-morph, the frequency of *Bombus* sp. visiting flowers was 0.14 ± 0.04 times/flower/hour, while the frequency of *Lasioglossum* sp. visiting flowers was 0.15 ± 0.05 times/flower/hour. There was no significant difference between the two morphs of visitors (Wald χ^2^ = 0.008, df = 1, *P* = 0.564); In the L-morph, there was no significant difference in the frequency of visiting flowers between the *Bombus* sp. (0.32 ± 0.11 times/flower/hour) and the *Lasioglossum* sp. (0.18 ± 0.02 times/flower/hour) (Wald χ^2^ = 0.931, df = 1, *P* = 0.435). Moreover, there were no significant differences (all *P* > 0.05) in the frequency of visits between the two morphs between the *Bombus* sp. and *Lasioglossum* sp. (**Figure 3**).

### Pollen germination and breeding system

**Figure 4:**
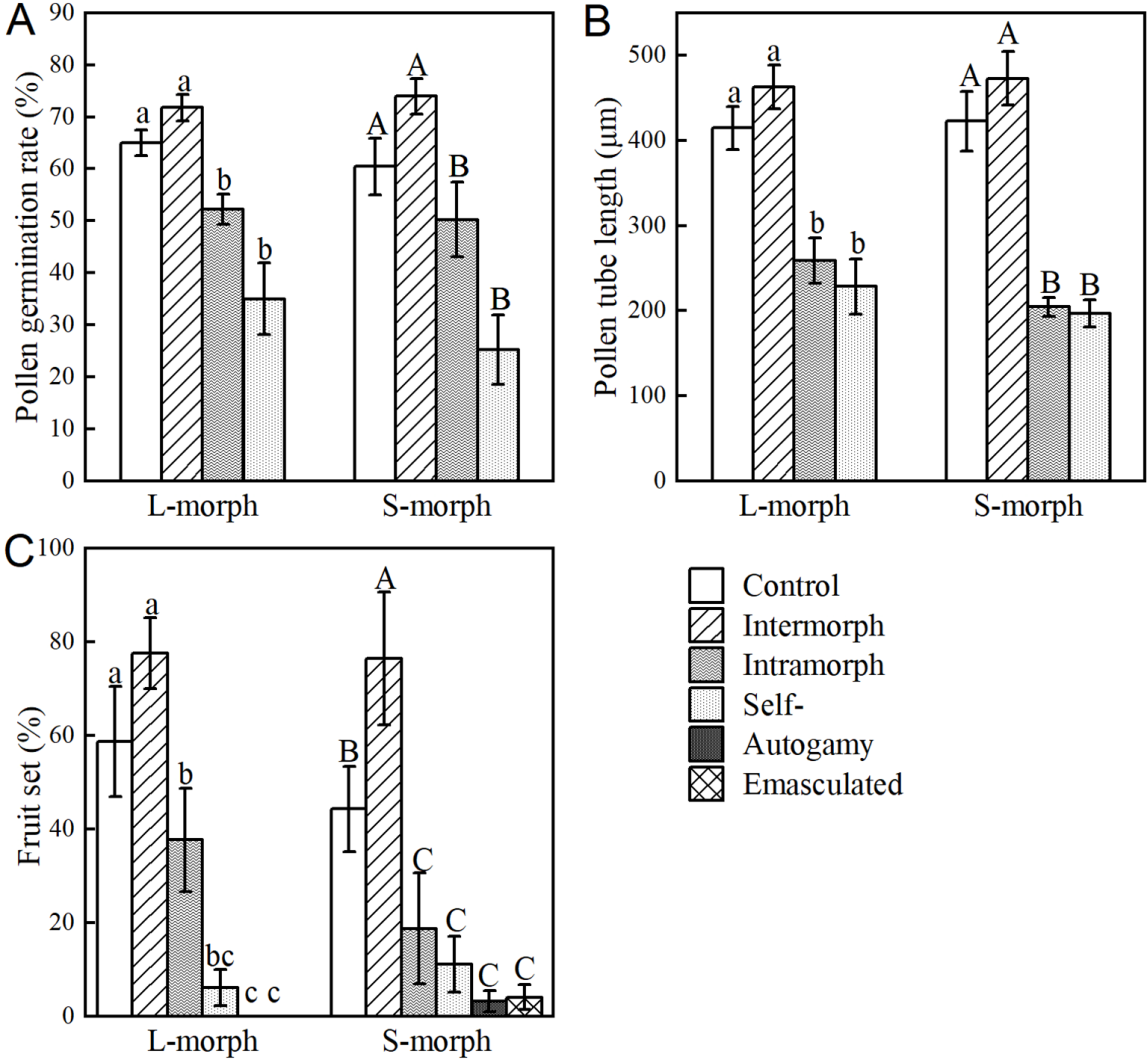
Pollen germination and fruit set of *O. alatiflora* under different pollination treatments. **(A)** Comparison of pollen germination rates under different pollination treatments in *O. alatiflora*. **(B)** Comparison of the elongation length of pollen tubes under different pollination treatments in *O. alatiflora*. **(C)** Comparison of the fruit set under different pollination treatments in *O. alatiflora*. Uppercase and lowercase letters represent different morphs, lowercase letters represent L-morphs, and uppercase letters represent S-morphs.

For the S-morph, the fruit set of intermorph outcrossing (76.42 ± 14.23%) was significantly higher than that of control (44.24 ± 9.05%) and other pollination treatments (Wald χ^2^ = 84.283, df = 5, *P* < 0.001), intramorph outcrossing (18.68 ± 11.86%), self-pollination (11.11 ± 5.92%), autogamy pollination (3.21 ± 2.22%), and emasculated pollination (4.07 ± 2.71%) treatments had significantly lower fruit set than that of control pollination treatments (Wald χ^2^ = 65.749, df = 4, *P* < 0.001). However, there was no significant difference between the fruit set of intermorph outcrossing (77.47 ± 7.59%) and the control (58.72 ± 11.80%) in the L-morph, and both were significantly higher than the fruit set of other pollination treatments (Wald χ^2^ = 125.202, df = 5, *P* < 0.001). The above results indicate that the fruit set of intramorph outcrossing between the two morphs of *O. alatiflora* is the highest (**Figure 4C**).

To further investigate the differences in affinity between different morphs, the pollen germination rates of different pollination treatments showed that in the S-morph, the germination rates of control (60.44 ± 5.45%), intermorph outcrossing (77.31 ± 1.96%), and intramorph outcrossing (50.26 ± 7.25%) pollen were significantly higher than those of self- pollen (25.24 ± 6.73%) (Wald χ^2^ = 55.894, df = 3, *P* < 0.001). In the L-morph, the pollen germination rates of intermorph outcrossing (73.93 ± 3.42%) were significantly higher than those of control (65.01 ± 2.48%) and other pollination treatments (Wald χ^2^ = 128.724, df = 3, *P <*0.001) (**Figure 4A**). The results of pollen tube elongation for different pollination treatments showed that the length of pollen tubes in S-morph intermorph outcrossing (376.24 ± 24.4 μm) was significantly greater than those of intramorph outcrossing (204.35 ± 11.21 μm) and self-pollination (300.17 ± 28.13 μm), while had no significant difference with control (219.36 ± 19.47 μm) (Wald χ^2^ = 42.585, df = 3, *P* < 0.001). The same was true in the L-morph, the length of pollen tubes intermorph outcrossing (462.73 ± 25.63 μm) was significantly greater than those of intramorph outcrossing (202.42 ± 11.08 μm) and self-pollination (151.59 ± 12.48 μm), while had no significant difference with control (207.94 ± 11.82 μm) (Wald χ^2^ = 218.875, df = 3, *P* < 0.001) (**Figure 4B**).

Significant interactions were found between pollination treatments and pollen recipient morph for germination rates. The pollen tube length and fruit set of different pollination treatments differed significantly and there was no interaction between pollination treatments and pollen recipient morph for pollen tube length and fruit set (**Table 1**).

**Table 1:** Effect of pollination treatments (control, intramorph pollination, intermorph pollination, and self-pollination), pollen recipient morph (L-morph vs. S-morph), and their interaction on pollen germination rates, pollen tube length, and fruit set in *O. alatiflora* (GLM).

Source of variation	Waldχ^2^	df	*P*
Germination rates (%)			
Pollination treatments	57.912	3	< 0.001
Pollen recipient morph	0.614	1	0.433
Interaction	8.497	3	0.037
Pollen tube length (μm)			
Pollination treatments	140.717	3	< 0.001
Pollen recipient morph	0.802	1	0.370
Interaction	2.098	3	0.552
Fruit set (%)			
Pollination treatments	47.965	5	< 0.001
Pollen recipient morph	0.660	1	0.416
Interaction	1.688	4	0.640

### Ancillary polymorphic

**Figure 5:**
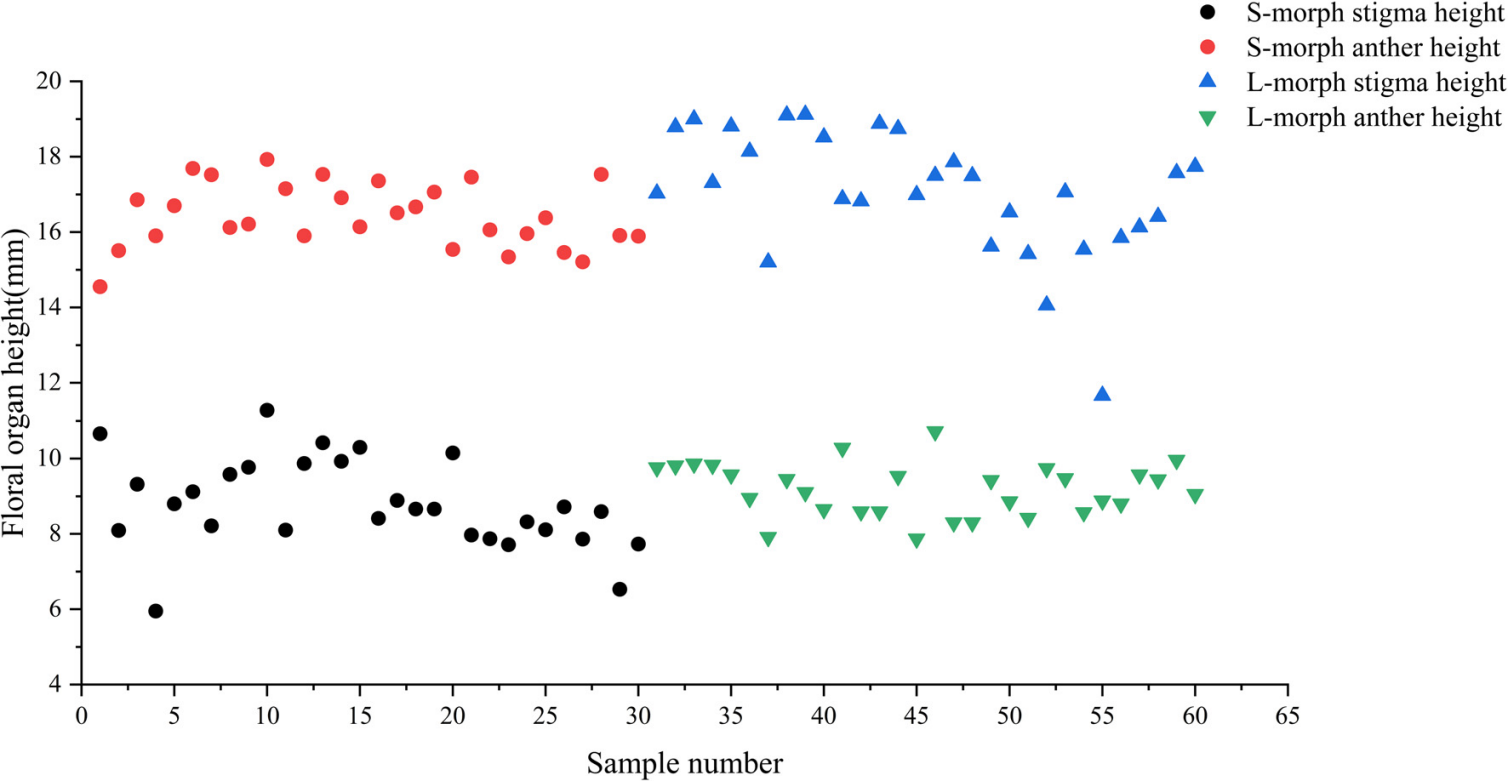
Height distribution of male and female organs in the two morphs.

For floral traits, the petal length, and petal width of the S-morph were significantly greater than those of the L-morph (all *P* < 0.05) (**Table 2**). There was no significant difference between pistil length of the L-morph (16.82 ± 0.39 mm) and stamen length of S-morph (16.43 ± 0.16 mm) (Wald χ^2^ = 0.835, df = 1, *P* = 0.361), pistil length of the S-morph (9.12 ± 0.38 mm) and stamen length of L-morph (9.42 ± 0.29 mm) were not significantly different (Wald χ^2^ = 0.391, df = 1, *P* = 0.532). The high-level sexual organ interaction index RH = -0.012, and the low-level sexual organ interaction index RL = 0.017, This indicates that the two morph sexual organs in *O. alatiflora* exhibit good interactivity (**Figure 5**).

**Table 2:** Comparisons of vegetative and reproductive traits (mean ± SE) between L-morphs and S-morphs of *O. alatiflora* tested by a generalized linear model (GLM) analysis.

Traits	L-morph	S-morph	Waldχ^2^	*P*
Leaf length (mm)	126.09 ± 3.29	126.04 ± 3.98	< 0.001	0.992
Leaf width (mm)	68.80 ± 17.76	51.65 ± 1.93	0.954	0.329
Flower length (mm)	19.16 ± 0.24	19.92 ± 0.34	3.311	0.069
Flower width (mm)	11.31 ± 0.30	12.09 ± 0.34	2.901	0.089
Petal length (mm)	5.09 ± 0.13	**5.72 ± 0.14**	10.892	0.001
Petal width (mm)	2.88 ± 0.08	**3.11 ± 0.06**	5.469	0.019
Tube depth (mm)	16.13 ± 0.20	16.66 ± 0.21	3.386	0.066
Tube diameter (mm)	2.94 ± 0.11	3.05 ± 0.16	0.302	0.582
Opening diameter (mm)	2.92 ± 0.10	2.94 ± 0.10	0.013	0.909
Pistil length (mm)	**16.82 ± 0.39**	9.12 ± 0.38	200.937	< 0.001
Stamen length (mm)	9.42 ± 0.29	**16.43 ± 0.16**	450.683	< 0.001
Anther length (mm)	2.95 ± 0.07	3.08 ± 0.06	2.043	0.153
Anther width (mm)	0.31 ± 0.02	0.33 ± 0.02	0.788	0.375
Anther thickness (mm)	0.29 ± 0.01	0.32 ± 0.02	1.785	0.182
Stigma length (mm)	2.59 ± 0.12	**3.20 ± 0.10**	15.12	<0.001
Stigma width (mm)	**0.55 ± 0.03**	0.38 ± 0.03	16.378	<0.001
Stigma thickness (mm)	0.43 ± 0.02	0.35 ± 0.02	7.343	0.007
Pollen grain number	**8983.33 ± 651.22**	5696.97 ± 251.71	5.165	0.023
Ovule number	110.45 ± 7.65	**142.64 ± 4.64**	11.715	0.001
Pollen/ovule ratio	**89.43 ± 8.43**	40.99 ± 2.22	30.875	0.001

Values for one morph that are significantly larger than the other are written in bold.

Statistics on the anthesis of *O. alatiflora* showed that the L-morph (2.07 ± 0.12 d) was significantly higher than that of (Waldχ^2^ = 4.698, df = 1, *P* = 0.030) S-morph (1.50 ± 0.27 d). Analysis of pollen and ovules of different morphs showed that the number of pollen in the L-morph was significantly higher than the S-morph (*P* < 0.05), and the number of ovules in the S-morph was significantly higher than the L-morph. The ratio of L-morph P/O is 89.43 ± 8.43, while the ratio of S-morph P/O is 40.99 ± 2.22. There was a significant difference in the ratio of L-morph and S-morph P/O (*P* < 0.001).

There was no significant difference in nectar volume and sugar concentration between the two morphs (*P* > 0.05).

### Secondary metabolites

**Figure 6:**
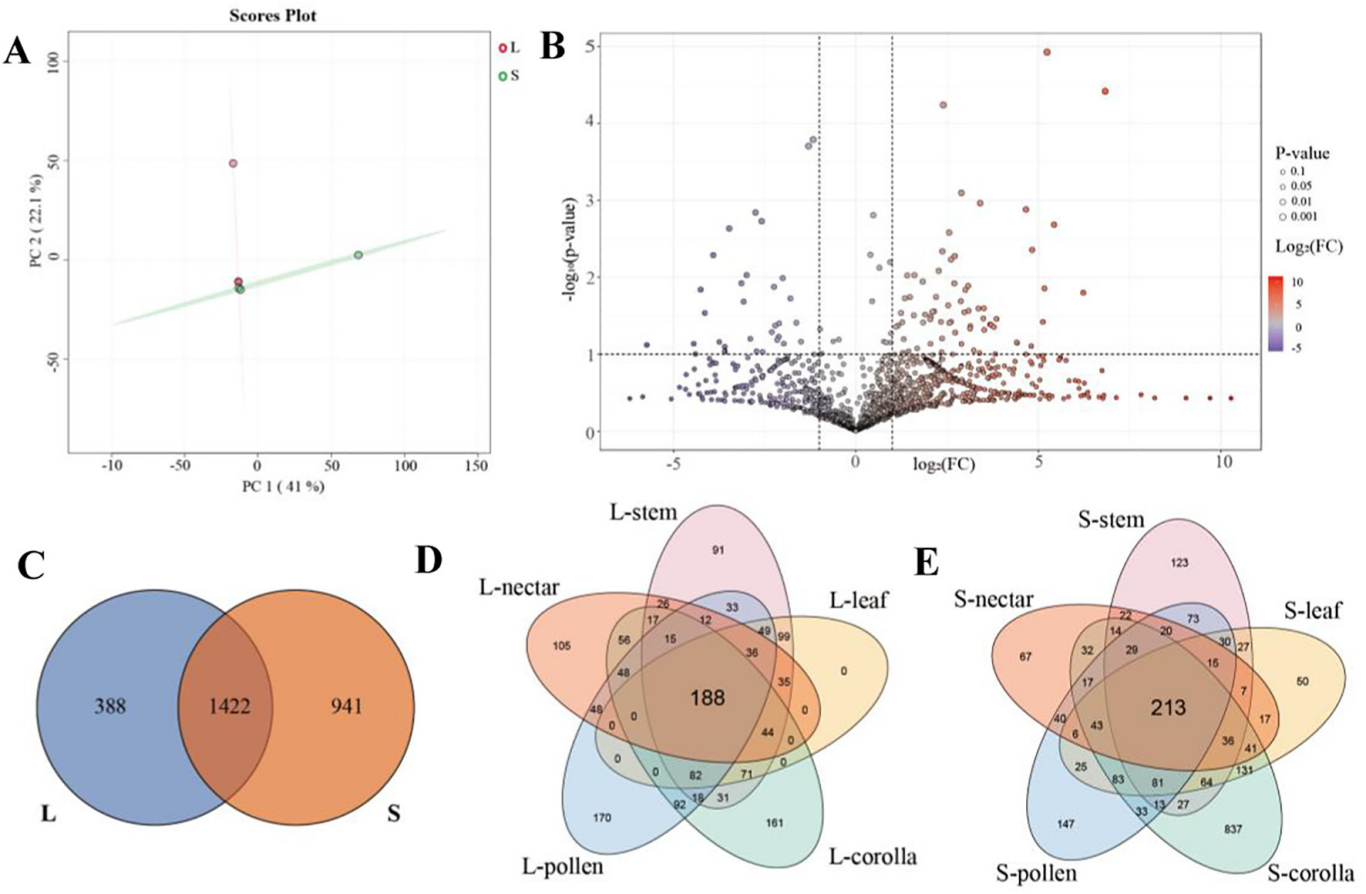
Differential analysis of secondary metabolites in *O. alatiflora*. **(A)** PCA analysis of secondary metabolites in L-morph and S-morph plants; **(B)** Volcano plot of secondary metabolite analysis in L-morph compared to S-morph, with purple representing downregulation and red representing upregulation; **(C)** Analysis of secondary metabolites shared by L-morph and S-morph; **(D)** Analysis of secondary metabolites shared by different tissues of L-morph plants; **(E)** Analysis of secondary metabolites shared by different tissues of S-morph plants.

For metabolome analysis, samples were examined using a broadly targeted LC-tandem MS (LC-MS/MS)-based metabolic profiling method. A total of 2, 751 distinct annotated metabolites were detected. Overall, there are 1422 substances in the L-morph and S-morph, accounting for 51.69% of the total substances (**Figure 6C**). Compared to the S-morph, the L-morph has 17 significantly downregulated metabolites, primarily flavonoids and terpenes, and 53 significantly upregulated differential metabolites, primarily alkaloids, and terpenoids (**Table 3**). From the perspective of tissue organs, there are 188 substances common to all five organ types in L-morph, compared to 213 substances in S-morph (**Figures 6D, E**). Additionally, significant differences exist in the types of secondary metabolites between tissues in L-morph and S-morph (**Supplementary Figure S1**). Notably, the leaf tissues of L-morph and S-morph show the greatest disparity in secondary metabolite types. Specifically, L-morph plants have 9 substances significantly downregulated and 34 substances significantly up-regulated, compared to S-morph plants (**Supplementary Table S2**). In contrast, the corollas of L-morph and S-morph exhibit minimal difference, with L-morph plants having 4 significantly downregulated substances and 3 significantly upregulated substances (**Supplementary Table S5**). Differences in metabolite types for other tissues and organs among the different morphs are detailed in **Supplementary Tables S2–S6** and **Supplementary Figures S1–S6**.

**Table 3:** Top ten differential secondary metabolites between L-morphs and S-morphs of *O. alatiflora* (L-morph vs. S-morph).

Category	Chemical compounds	Molecular	log_2_(FC)	VIP	P-values	Expression trend
Flavonoids	Cpinme F	C_30_H_22_O_10_	-4.252	1.978	0.015	down
Flavonoids	Baohuoside I	C_27_H_30_O_10_	-4.139	1.877	0.029	down
Flavonoids	Delphinidi	C_15_H_14_O_8_	-3.909	2.049	0.005	down
Terpenoids	Paeonenolide H	C_31_H_48_O_5_	-3.472	2.099	0.002	down
Triterpenoids	Raddeanoside D	C_47_H_76_O_16_	-3.137	1.980	0.012	down
Flavonoids	5,7-Dihydroxychromone-7-β-D-glucopyranoside	C_15_H_16_O_9_	-3.075	1.915	0.021	down
Phenols	1-Hydroxy-2-methylanthraquinone	C_15_H_10_O_3_	-2.993	2.000	0.009	down
Terpenoids	Betulinic acid methyl ester	C_31_H_50_O_3_	-2.752	2.123	0.001	down
Flavonoids	Baohuoside II	C_26_H_28_O_10_	-2.582	2.110	0.002	down
Others	Emodin	C_15_H_10_O_5_	-2.244	1.983	0.013	down
Triterpenoids	Poricoic acid AM	C_32_H_48_O_5_	6.826	2.175	<0.001	up
Terpenoids	Prosapogenin 7 (Julibroside A3)	C_49_H_77_NO_17_	6.227	1.964	0.016	up
Terpenoids	Borneol-2-O-glucopyranoside	C_16_H_28_O_6_	5.425	2.100	0.002	up
Terpenoids	Nigakilactone E	C_24_H_34_O_8_	5.232	2.179	<0.001	up
Others	3-O-β-D-xylopyrano-(1→2)-α-L-arabinose pyranos hederin	C_40_H_64_O_12_	5.162	1.976	0.014	up
Terpenoids	Paeoniflorin	C_23_H_28_O_11_	5.117	1.821	0.038	up
Steroid	Marsdenoside A	C_45_H_70_O_14_	4.826	2.063	0.004	up
Triterpenoids	Asetr saponin Hb	C_48_H_76_O_18_	4.656	2.125	0.001	up
Triterpenoids	CurculigosaPonin H	C_47_H_78_O_17_	3.847	1.837	0.035	up
Phenols	Aschantin	C_21_H_26_O_6_	3.775	1.788	0.044	up

## Discussion

### The incomplete self-incompatibility system of *O. alatiflora* is regulated by both pollen affinity and male-female ectopia

Darwin (1877) proposed that the spatial heterotopy of male and female organs in heterostyled plants can effectively promote the legal transmission of pollen between different morphs, but heterostyly cannot be considered as the only evolutionary source to avoid self-pollination and promote outcrossing (Zhou and Wang, 2009) Studies have shown that the self-incompatibility system of plants is controlled by a pair of key genes at the physiological S locus, and the incompatible system of heterostyled not only prevents self-pollination but also reduces the success rate of intramorph outcrossing. Therefore, heterostyly as a mechanism to promote outcrossing is probably inefficient (Ren et al., 2004; Barrett and Shore, 2008). Other viewpoints suggest that the evolution of heteromorphic styles was to reduce male-female interference, for example, the study of the characteristics of the distyly of *Forsythia suspensa*, and the dimorphic style of *Primula obconica* (Beach and Bawa, 1980; Dulberger, 1992; McCall, 1996; Sun et al., 2021). However, there are also reports that heterostyly does not significantly help in avoiding male and female ectopia (Barrett and Glover, 1985). In summary, the evolution of heterostyly systems is very complex.

Our study reports a newly discovered heterostyled plant, *O. alatiflora*, and tested the breeding system of *O. alatiflora* by the fruit set of pollination treatments. Research shows that the fruit set of intermorph outcrossing is highest among five manual treatments both in L-morph and S-morph, and partial self-compatibility and intramorph outcrossing compatibility is discovered in both two morphs, the heterozygous incompatible system does not always function strictly like the typical self-compatibility controlled by the S locus. The precise alignment of the stigma and anthers of the *O. alatiflora* effectively reduces the deposition of illegal pollen by isolating pollen on pollinators, improves intermorph pollination efficiency, promotes cross-pollination in physical space, and avoids contact between self-pollen or intramorph pollen as much as possible. Meanwhile, although there was a phenomenon in our study where outcrossing between different morphs was much higher than self-crossing, it cannot be concluded that it was entirely caused by the ectopia of the two morphs in *O. alatiflora*. We tested the pollen affinity under different treatments through pollen tube germination and pollen tube elongation experiments. The results showed that the changes in fruit set under different treatments were almost consistent with pollen affinity, indicating that another major factor causing high outcrossing and low self-crossing in *O. alatiflora* may be the physiological affinity of pollen itself, that is, intermorph pollen affinity is high, while self or intra pollen affinity is low. Therefore, the breeding system of *O. alatiflora* is influenced by the physiological affinity of pollen and the precise ectopia of male and female organs in physical space, ultimately exhibiting a high success rate of intermorph outcrossing and maintaining rich genetic diversity. At the same time, there is a small amount of intramorph and self-pollination success power to ensure the reproduction of its species. This may be a unique breeding strategy for the *O. alatiflora* in fragmented habitats.

Previous studies have reported that some distylous plants in karst areas exhibit similar reproductive strategies, such as *Luculia pinceana* (Chen et al., 2021) and *Ceratostigma willmottianum* (Gao et al., 2021), both of which demonstrate high heterocompatibility and a limited degree of self-compatibility. In contrast, *Tirpitzia sinensis* exhibits a distinct male-female phenomenon and adheres to a strict self-incompatibility system, potentially due to its specialized pollinators and high pollination efficiency (Hu et al., 2021). Currently, the reproductive strategies of distylous plants in karst areas are influenced by habitat fragmentation; however, it is essential to consider additional factors, such as pollinator types and pollination methods. More comprehensive studies are needed to determine whether habitat fragmentation in karst areas predominantly influences the selection of reproductive strategies in dimorphic plants.

### The differential ancillary polymorphic between the two morphs improved their respective male and female fitness

The flower morphology of *O. alatiflora* presents a cylindrical shape with herkogamy. The interaction index indicated that different morphs of male and female organs have reciprocal stigma-anther, consistent with the characteristics of distylous species, which was also observed in plants of the Rubiaceae family (Barrett, 2000). For insect pollinator plants, herkogamy greatly affects the location of pollen carried on the insects’ bodies and the position of pollen deposit on the stigma. The precise complementarity of stigma-anther reciprocity is conducive to the occurrence of outcrossing (Barrett, 1990; Lloyd, 1992). Darwin’s (1877) initial definition of heterostyled plants encompassed various traits. However, subsequent research revealed that these characteristics do not consistently exhibit significant variations among different morphs, and even among heterostylous species within the same genus, polymorphism may occur. Consequently, these traits are not essential for defining heterostylous plants (Zhou and Wang, 2009). At present, it is widely believed that complementary herkogamy is a classic feature of heterostyled plants, while other features between different styles are ancillary polymorphic (Chen et al., 2010). In the early stage, technology limited researchers’ further exploration of ancillary polymorphic, ancillary polymorphic in stamen characteristics, such as the number and size of pollen grains, the ornamentation of the pollen exine, and the size and color, of anthers as well as stigma depth and receptive area. As technology has advanced, an increasing number of plant secondary metabolites have been identified as playing crucial roles in regulating plant behavioral functions. In this study, we propose expanding the concept of ancillary polymorphic to include variations in plant secondary metabolites. This approach will enable a more comprehensive analysis of the complexities associated with heterostylous plants.

L-morph flowers, due to their exposed higher stigma, are more likely to receive crossing pollen and have higher female fitness compared to hidden S-morph flowers. The anthers of S-morph flowers are higher than the thrum stigma, making their pollen more easily removed by pollinators and exhibiting higher male fitness. On the other hand, some researchers have pointed out that S-morph flowers are at risk of self-pollination because the stigma is hidden under the anthers. Our results on pollination show that the self-pollination setting rate of S-morph flowers is indeed slightly higher than that of L-morph flowers, but fruit set in open pollination is higher than self-pollination for both morphs flowers, indicating that the presence of pollinators plays an important role in the reproduction of *O. alatiflora* in both L-morph and S-morph flowers. Meanwhile, in open pollination, the fruit set of L-morph flowers is higher than that of S-morph flowers, indicating that L-morph has higher female fitness.

In the plants of *O. alatiflora*, the number of L-morph pollen is significantly higher than that of S-morph, while the number of ovules is significantly lower than that of S-morph pollen, which may compensate for the asymmetry of pollen flow (Ganguly and Barua, 2020). It is speculated that the L-morph of *O. alatiflora* produces more pollen to compensate for the weak male function and increase pollen removal during pollinator visits, while the S-morph tends to produce more ovules to enhance their female fitness. In addition, many studies have found that the corolla size of L-morph is smaller than that of S-morph. Some viewpoints suggest that S-morph flowers that rely on insect pollination have a lower chance of obtaining pollen because the stigma is hidden inside the flower compared to L-morph flowers with the stigma exposed outside the corolla. S-morph flowers are larger to provide more opportunities for insect pollination (Dulberger, 1992; Chen et al., 2010). Our results are consistent with the previous study. In a word, the differences in ancillary polymorphic between two morphs of *O. alatiflora* improved their male and female fitness respectively, thus promoting effective pollination.

### Differences in secondary metabolites of different morphs in *O. alatiflora*

The species of the *Ophiorrhiza* genus have been associated with claims of various medicinal properties and wide-ranging applications in traditional and modern medicine alike (Sibi et al., 2012; Midhu et al., 2019; Sahukari et al., 2021). The whole plant is rich in active compounds, including flavonoids, terpenes, and alkaloids. Its Chinese name means snake-like root herb. Associated with its name, the *Ophiorrhiza* species are capable of healing snakebite, stomatitis, ulcers, and wounds (Midhu et al., 2019; Sahukari et al., 2021), while also acting as an antioxidant (Martins and Nunez, 2015), antitussive, and analgesic alternative (Sibi et al., 2012). As an endemic species in China, the chemical compounds of *O. alatiflora* have not been determined. Harmine is found in the *Ophiorrhiza* genus, which is a β-carboline alkaloid with various pharmacological activities, including antioxidant, anti-inflammatory, and antitumor capabilities (Palmer-Young et al., 2019).

In this study, S-morphs exhibited a greater variety of secondary metabolites, with 603 more than the L-morph. However, not all of these substances displayed significant differences (**Figure 6**). Analysis revealed that L-morphs had significantly higher levels of some terpenoids, compared to S-morphs, while S-morphs produced more flavonoids than L-morphs (**Table 3**; **Supplementary Table S1**). Terpenoids are the most abundant volatile organic compounds (VOCs) in plants, with over 80,000 known species (Christianson, 2017). Terpenes are crucial secondary metabolites, involved in regulating plant growth, development, pollination, resistance to biotic or abiotic stress, and photosynthesis (Loreto et al., 2014; Wang et al., 2019). Some terpenoids attract insects for pollination or participate in plant-induced defense responses due to their fragrance and taste (Taniguchi et al., 2014; Gao et al., 2015; Chen et al., 2018; Wang et al., 2019b; Aartsma et al., 2020; Sun et al., 2022). For instance, the monoterpene β-ocimene is a common plant volatile that serves various biological functions, from a generalist attractant of a wide spectrum of pollinators to mediating defensive responses to herbivory (Farré-Armengol et al., 2016, 2017; Ramya et al., 2020). The content of Poricoic acid AM, Prosapogenin 7 (Julibroside A3), Borneol-2-O-glucopyranoside, and Nigakilactone E in L-type plants of *O. alatiflora* is 113 times, 74 times, 42 times, and 37 times higher than that in S-morphs, respectively (**Table 3**, **Supplementary Table S1**), suggesting that L-morphs may have unique mechanisms for attracting flower visitors or resist herbivores compared to S-morphs. Additionally, flavonoids are significant secondary metabolites in plants, often linked to the regulation of flower color, which is crucial for attracting insects and birds for pollination, which helps plants survive. Although 400 – 500 anthocyanins have been reported, six anthocyanidins, i.e., pelargonidin, cyanidin, peonidin, delphinidin, petunidin, and malvidin, are common in nature. Among these, is delphinidin, in which hydroxyl groups are attached to the 3’ -, 4’-, and 5’ - positions in the B ring, therefore plants containing more delphinidin tend to be blue-green (Iwashina, 2015). In *O. alatiflora*, S-morphs have significantly higher levels of delphinidin than those in L-morphs (**Table 3**), yet phenotypically, S-morphs are not visibly bluer but similar to L-morphs, both appearing purple-red or white. For instance, *Linum grandiflorum* (Linaceae) also contains a high level of delphinidin but exhibits scattered flowers blue, which may be the result of the combined action of other flavonoids (Toki et al., 1995). In addition, the content of Cpinme F and Baohuoside I in S-morphs of the *O. alatiflora* is 20 and 7 times higher than that in L-morphs, respectively, indicating that S-morphs have considerable potential for producing flavonoids (**Table 3**).

To investigate the differences in secondary metabolites between different tissues in *O. alatiflora*, we performed a differential metabolite analysis. The results revealed that the primary differences in secondary metabolites between L-morphs and S-morphs were observed in the nutritional organs such as stems and leaves. Conversely, the corolla of the reproductive organ exhibited minimal variation, with only six differential metabolites identified between the corollas of the two morphs (**Supplementary Tables S2–S6**). It remains unclear whether these differential metabolites contribute to different phenotypic or functional traits, indicating a need for further research. Despite L-morphs and S-morphs being the same species, they produce different types and quantities of secondary metabolites, and notable differences in these secondary metabolites exist between stems and leaves within the same morph. These variations may be linked to functional traits, such as certain terpenoids aiding in pollinator attraction or alkaloids serving as deterrents to herbivores. The functions of the differentially expressed substances identified in this study are not well-documented. Further research will aim to uncover valuable substances and provide insights into the medicinal potential of *O. alatiflora*.

## Conclusion

In this study, we report a new distylous plant, *O. alatiflora*, that grows in karst areas, and explore its reproductive strategy in fragmented habitats. The research indicates that the herkogamy of *O. alatiflora* is precisely reciprocal, but it has an incomplete self-incompatibility system. The precise herkogamy of the *O. alatiflora* facilitates the plant to gain effective intermorph pollen flow in space and avoids receiving ineffective self- or intramorph pollination, this reproductive strategy is similar to some of the reported distylous plants growing in karst areas, including *Luculia pinceana* (Chen et al., 2021) and *Ceratostigma willmottianum* (Gao et al., 2021). This may be the result of plant convergent evolution. At the same time, there are still some distylous plants growing in karst areas that have the opposite reproductive strategy to *O. alatiflora*. For example, the abundance of specialized pollinators in *Tirpitzia sinensis* may partially compensate for the impact of habitat fragmentation (Hu et al., 2021). Overall, the large-scale habitat fragmentation in karst may have to some extent affected the less strict adherence to self-incompatibility in distylous plants. However, whether this factor dominates requires more comprehensive research to determine, including the interaction between plants and pollinators, as well as the interaction between plants and the environment. At the same time, the different physiological affinities of pollen also lead to a high intermorph outcrossing rate in *O. alatiflora* and even maintain a partially self-compatible breeding system, which could ensure genetic diversity and the reproduction of this species, this may be one of the adaptation strategies in fragmented habitats. In addition, the auxiliary characteristics of flower morphology in *O. alatiflora* exhibit polymorphism among different morphs. According to our investigation, L-morph flowers have obvious female functions. To compensate for male function, L-morph flowers produce more pollen and fewer ovules compared to S-morph flowers, while S-morph flowers produce the opposite. This reflects the differences in resource allocation strategies for different morphs. L-morphs exhibit significantly higher levels of certain terpenoids compared to the S-morphs. Conversely, S-morphs produce a greater quantity of flavonoids than L-morphs. Differences in secondary metabolites between L-morphs and S-morphs are most pronounced in the nutritional organs, such as stems and leaves, while the reproductive organs, particularly the corolla show minimal variation in the secondary metabolite types. The analysis of secondary metabolites in different morphs of *O. alatiflora* provides valuable insights, broadening the scope of ancillary polymorphic studies and offering a novel research approach for understanding the differences among morphs in heterostylous plants.

## Data Availability

The original contributions presented in the study are included in the article/**Supplementary Material**. Further inquiries can be directed to the corresponding authors.
