# Supplementary material for: The reproductive strategy of a typical distylous Ophiorrhiza alatiflora (Rubiaceae), in fragmented habitat
Source: Front Plant Sci. 2024 Nov 4;15:1492402. doi: 10.3389/fpls.2024.1492402 (PMC11570894; doi:10.3389/fpls.2024.1492402)
Supplement: Supplementary file 1 [file DataSheet1.pdf]

# *Supplementary Figures*

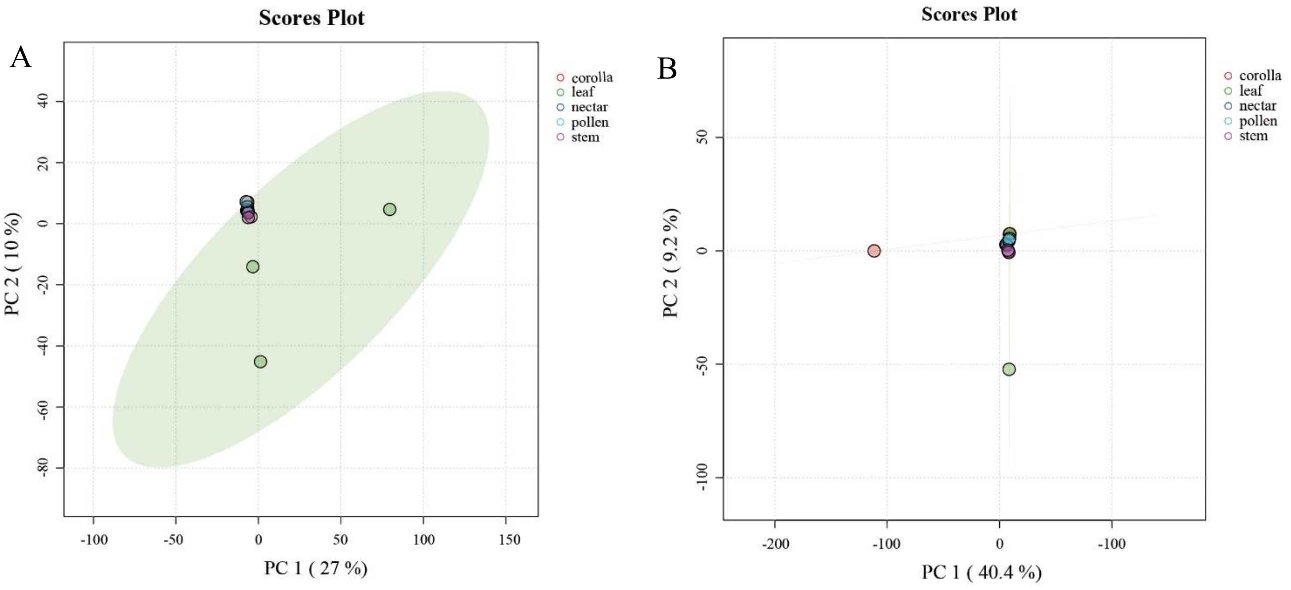


**Supplementary Figure 1** Differential analysis of secondary metabolites in different tissues of *O. alatiflora*. (A) PCA analysis of secondary metabolites in different tissues of L-morph plants. (B) PCA analysis of secondary metabolites in different tissues of S-morph plants.

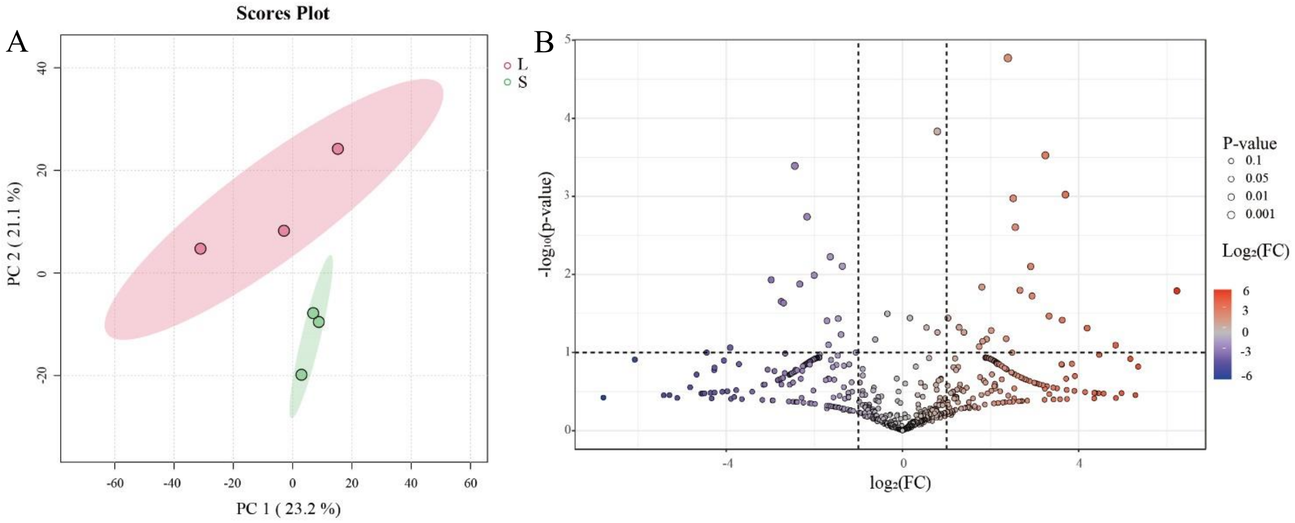


**Supplementary Figure 2** Differential analysis of secondary metabolites in stems of different morphs of *O. alatiflora*. (A) PCA analysis of secondary metabolites in stems of different morphs; (B) Volcanic plots of secondary metabolites in stems of different morphs, with purple representing downregulation and red representing upregulation.

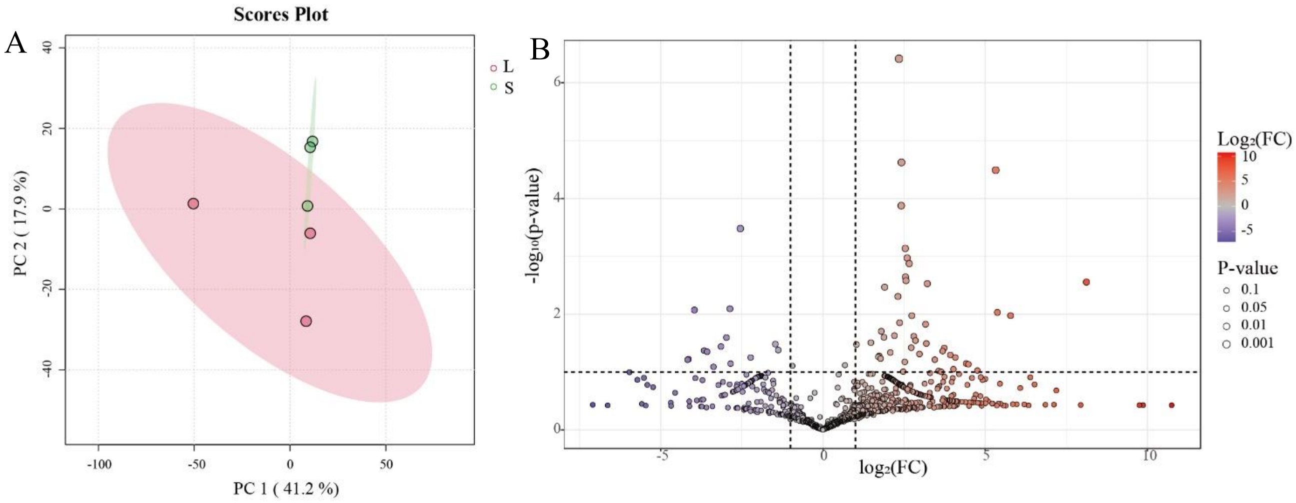


**Supplementary Figure 3** Differential analysis of secondary metabolites in leaves of different morphs of *O. alatiflora*. (A) PCA analysis of secondary metabolites in leaves of different morphs; (B) Volcanic plots of secondary metabolites in leaves of different morphs, with purple representing downregulation and red representing upregulation.

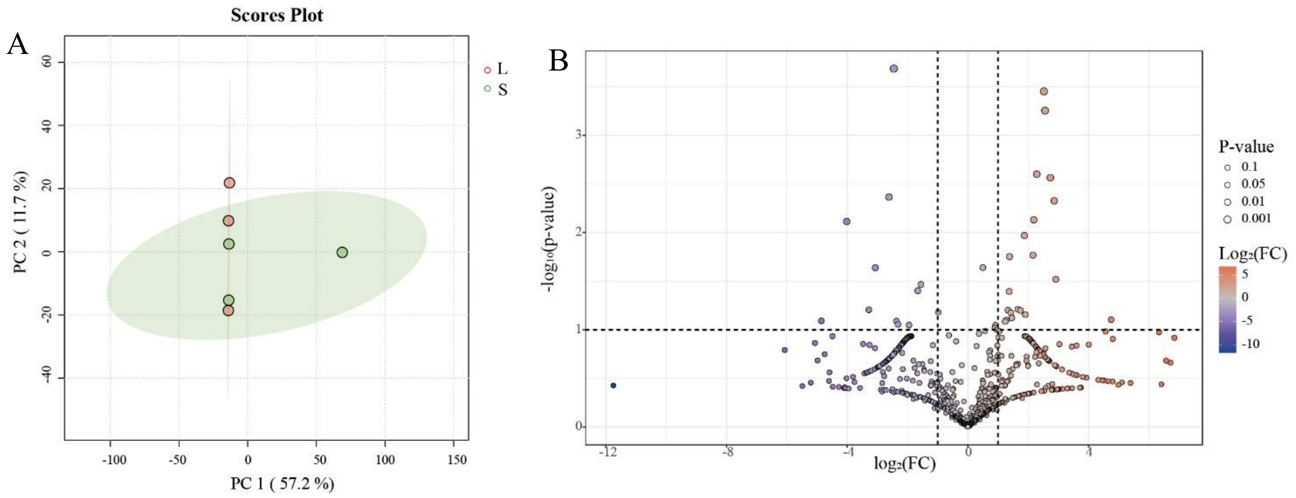


**Supplementary Figure 4** Differential analysis of secondary metabolites in the corolla of different morphs of *O. alatiflora*. (A) PCA analysis of secondary metabolites in the corolla of different morphs; (B) Volcanic plots of secondary metabolites in the corolla of different morphs, with purple representing downregulation and red representing upregulation.

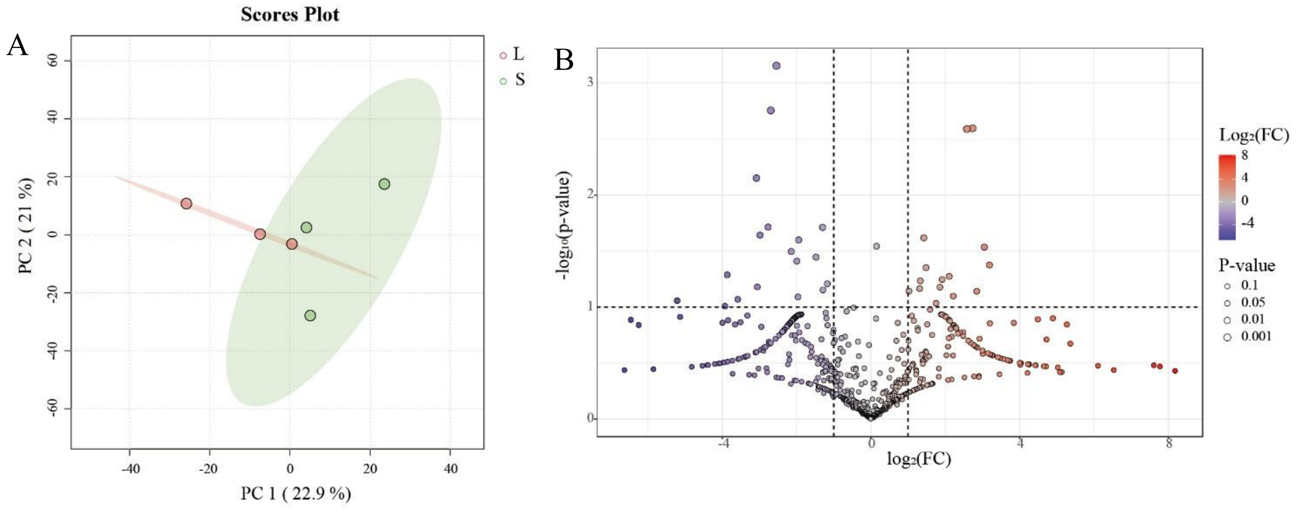


**Supplementary Figure 5** Differential analysis of secondary metabolites in pollen of different morphs of *O. alatiflora*. (A) PCA analysis of secondary metabolites in pollen of different morphs; (B) Volcanic plots of secondary metabolites in pollen of different morphs, with purple representing downregulation and red representing upregulation.

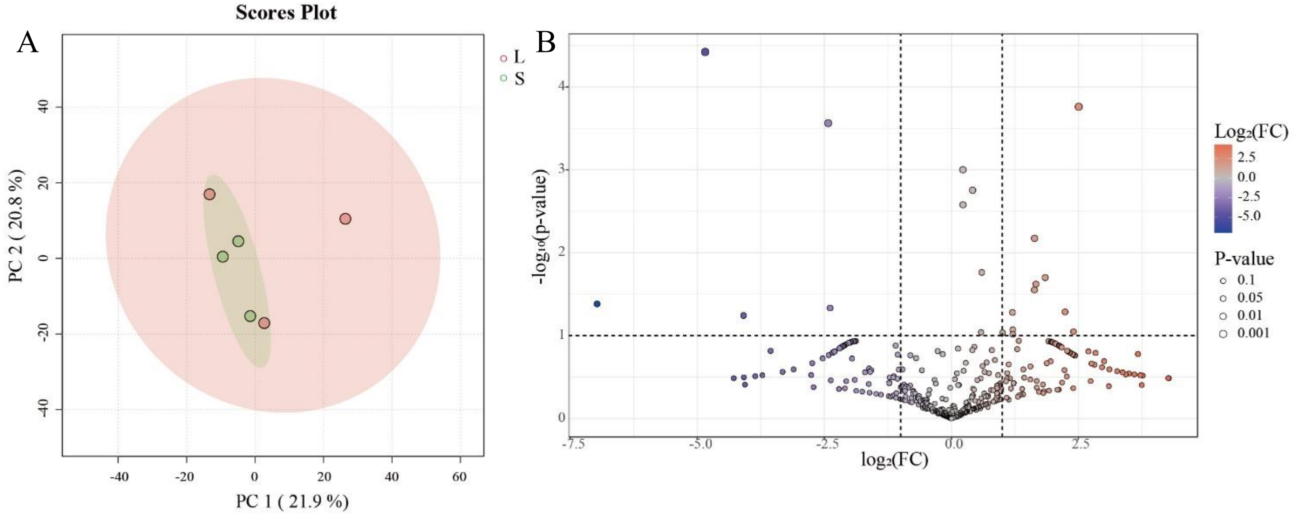


**Supplementary Figure 6** Differential analysis of secondary metabolites in the nectar of different morphs of *O. alatiflora*. (A) PCA analysis of secondary metabolites in the nectar of different morphs; (B) Volcanic plots of secondary metabolites in the nectar of different morphs, with purple representing downregulation and red representing upregulation.
